# The Wide and Complex Field of NAFLD Biomarker Research: Trends

**DOI:** 10.1155/2014/846923

**Published:** 2014-04-28

**Authors:** Erika Wichro, Tanja Macheiner, Jasmin Schmid, Barbara Kavsek, Karine Sargsyan

**Affiliations:** ^1^BioPersMed/Biobank Graz, Medical University of Graz, Stiftingtalstrasse 3.1, Graz, 8010 Styria, Austria; ^2^Siemens AG Austria, Corporate Technology, Biosensors, Strassganger Strasse 315, Graz, 8045 Styria, Austria

## Abstract

*Background*. Nonalcoholic fatty liver disease is now acknowledged as a complex public health issue linked to sedentary lifestyle, obesity, and related disorders like type 2 diabetes and metabolic syndrome. *Aims*. We aimed to retrieve its trends out of the huge amount of published data. Therefore, we conducted an extensive literature search to identify possible biomarker and/or biomarker combinations by retrospectively assessing and evaluating common and novel biomarkers to predict progression and prognosis of obesity related liver diseases. *Methodology*. We analyzed finally 62 articles accounting for 157 cohorts and 45,288 subjects. *Results*. Despite the various approaches, most cohorts were considerably small and rarely comparable. Also, we found that the same standard parameters were measured rather than novel biomarkers. Diagnostics approaches appeared incomparable. *Conclusions*. Further collaborative investigations on harmonizing ways of data acquisition and identifying such biomarkers for clinical use are necessary to yield sufficient significant results of potential biomarkers.

## 1. Introduction 

Nonalcoholic fatty liver disease (NAFLD) is acknowledged as a (public) health issue with an estimated prevalence of 30% in adults [1], of which approximately 25% progresses to nonalcoholic steatohepatitis (NASH) [2]. Sedentary lifestyle and high-fat and high-caloric dietary intake are strongly associated with NAFLD and nonalcoholic steatohepatitis (NASH). NAFLD and NASH occur not only in adults but also increasingly in childhood [3–5], accounting for a tremendous economic health burden [6].

The pathways of NAFLD/NASH and their alcohol-induced counterpart diseases are multifactorial, involving the liver metabolism key players: cytokines, adipokines, and apoptosis [7]. Alternative tools such as ultrasound or magnetic resonance imaging (MRI) [8] are becoming common in clinical routine; anyway in NAFLD/NASH diagnostics liver biopsy still remains the golden standard [9, 10].

In the context of personalized medicine, the research on biomarker to identify NAFLD and its development and progression is high priority for clinical routine. Our systematic data analysis of NAFLD research conducted in the last year, based on evaluation of extensive literature search, provided an overview of the most commonly published potential biomarkers for NAFLD.

## 2. Methodology 

**Figure 1:**
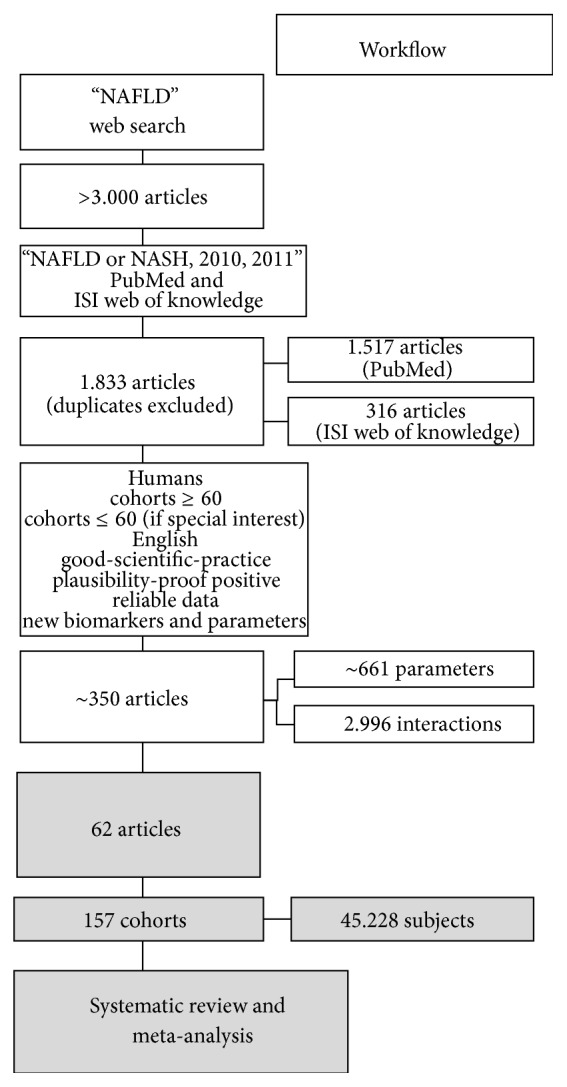
Workflow diagram.

We performed an extensive literature assessment to evaluate known and novel biomarkers for progression and prognosis of NASH/NAFLD. In this context, we extracted and evaluated cohorts and parameters and consolidated scientific findings from the literature. We limited our keywords to “NAFLD OR NASH, 2010, 2011,” accounting for a total of 1,833 articles, 1,517 articles from PubMed and 316 articles from ISI web of knowledge (Figure 1).

Cohorts meeting the selection criteria were included:

- english language;
- human subjects;
- adults;
- cohort size >60 subjects;
- sufficient and reliable data;
- good scientific practice;
- positive plausibility proof;
- balanced grouping;
- proven diagnosis (was not performed by an invasive liver biopsy at all studies).

Those cohorts not meeting the criteria were nevertheless scanned for potential parameters. Studies with cohorts below 60 subjects and presenting rare parameters such as potential biomarkers (e.g., ferritin, adiponectin, or rarely documented diabetic probands of NAFLD studies) and meeting all other criteria were included.

We transferred the cohorts of included studies in our subtypes' classification according to the definitions listed in Table 1.

## 3. Statistical Evaluation

Data-analysis approach is based on several requirements including studies with paired controls and balanced cohort size. The plots require an identical statistical summary measure, which means either using only the mean or only the median and requires the standard deviation known to derive confidence intervals. The information on deviations ranges or standard deviations (SD) proved not to be useful for further analysis and was omitted. To analyze a larger set of publications and to overcome limits of balanced cohort size, we included studies that had no paired control and pooled cohorts in several studies to ensure a comparable set.

The extracted mean values are displayed in boxplots by exploratory data analysis (EDA) and show median and 25 and 75 percentiles [11]. Mann-Whitney test was applied to compare the values between diseased and control groups; Kruskal-Wallis test was used to compare subjects of more than two groups. Statistical analysis was performed in R version 2.13.1 (R Foundation for Statistical Computing, Vienna, Austria). *P* values <0.05 were considered statistically significant.

Though a graphical representation of a small number of observations can be performed, a correct analysis is not possible. Therefore, statistical interpretation of results was performed particularly cautiously in such cases.

Finally, we used boxplots to visualize the measurements in arithmetic mean and the comparison between disease and relevant control groups.

## 4. Results and Discussion 

### 4.1. Results

Based on the inclusion criteria, we comprised 62 studies, accounting for 157 cohorts and 45,228 subjects. Overall, we evaluated 81 different parameters provided in the included studies. Additionally, we bridged lifestyle and biomarkers by also including anthropometric values. Including gender was impossible because most studies showed no gender separation, but fulfilled all required criteria.

**Figure 2:**
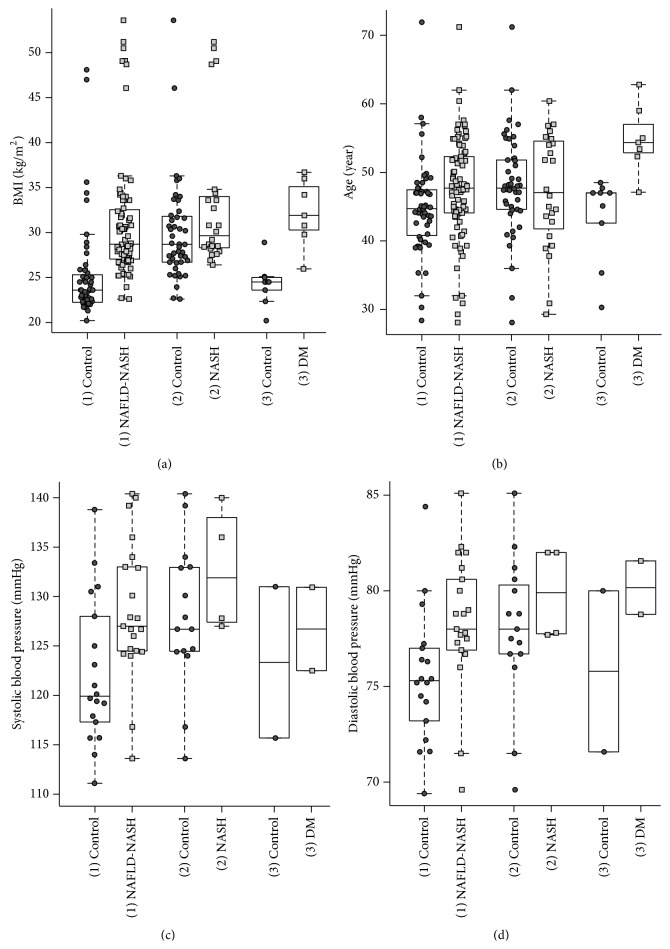
Basic parameters of analyzed studies. Relationship of BMI, age, SBP, and DBP between different disease groups and their controls. (a) BMI depicts significant differences between the control groups and their related disease groups, presenting the lowest values among the (1) control group containing healthy subjects, respectively. (b) Age presented higher results in the (1) NAFLD-NASH and the (3) DM groups than in their controls. ((c)-(d)) Both, systolic blood pressure (SBD) and diastolic blood pressure (DBP) depict an increase with the degree of NAFLD.

**Figure 3:**
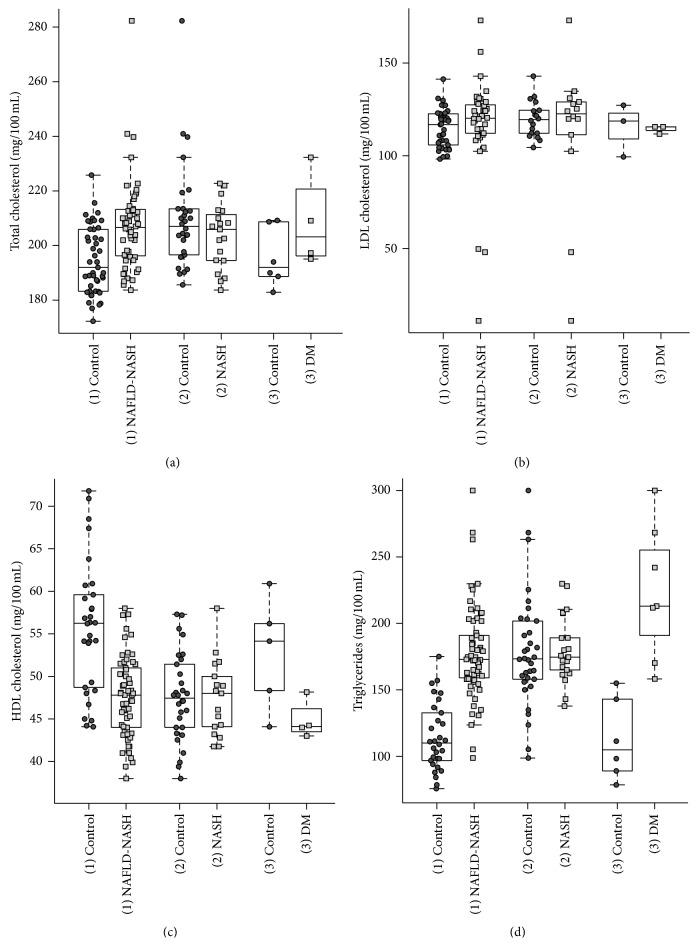
Lipid status analysis. Relationship of total cholesterol, LDL, HDL, and triglycerides between the disease groups and their controls. (a) The (1) NAFLD-NASH group presents significant higher results as its control group. (c) HDL appears higher in the (1) control group. ((a)–(d)) Merely, the triglycerides depict significant higher results in the NAFLD-NASH and the (3) DM group as compared to their controls.

**Figure 4:**
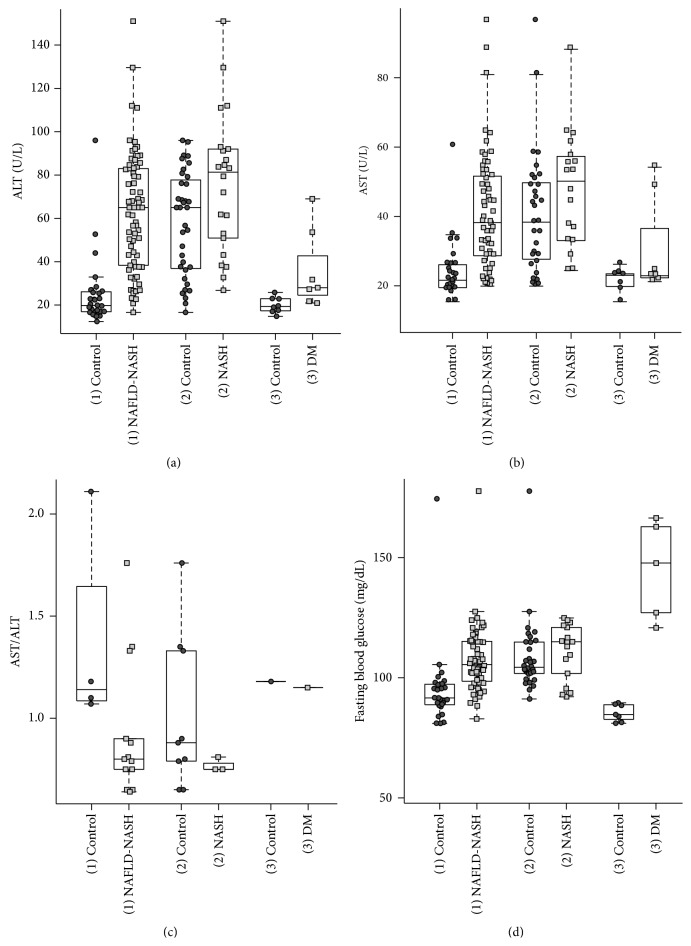
Liver enzymes analysis. Relationship of ALT, AST, AST/ALT, and fasting blood glucose (FBG) between the (1) NAFLD-NASH, the (2) NASH, the (3) DM groups, and their related control groups. ((a)–(d)) Overall, the parameters depict an increase in relation to the degree of NAFLD.

**Figure 5:**
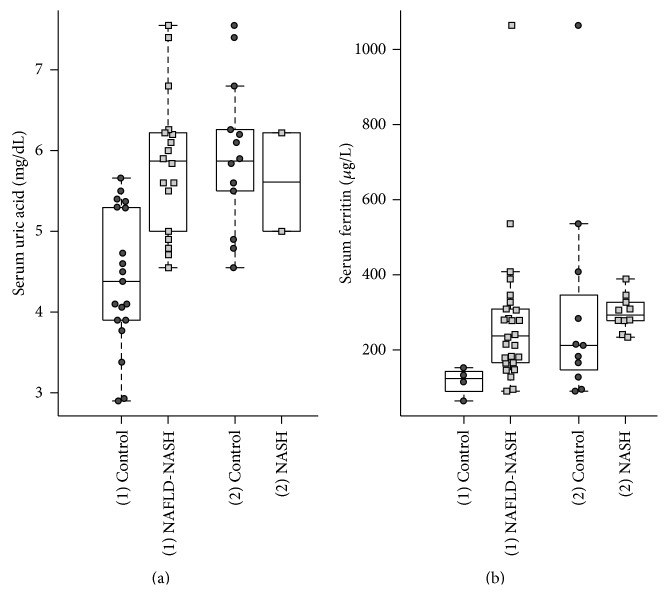
Promising parameters—potential novel biomarkers? Serum uric acid (SUA) and serum ferritin considered as potential biomarkers in the detection of NAFLD. ((a)-(b)) Both parameters illustrate significant difference between the disease groups and their controls.

The noteworthy results are summarized in Figures 2, 3, 4, and 5. The 62 included studies are listed in Table 2 and depicted results of different parameters given in arithmetic mean (75.8%), in median (11.3%) or mixed in mean and median (12.8%).

In our scope of evaluation, we focused on the analysis of (1) available data, (2) significant results, and (3) potential use of parameters.

Our data analysis considered conventional parameters such as BMI, age, and systolic and diastolic blood pressure (Figure 2).

The BMI results of the healthy control group (1) were significantly lower than those of different disease groups, whereby the BMI appeared also significantly lower in (1) control group than in the related NAFLD-NASH group (*P* value: 7.8 × 10^−12^). Likewise, a significant BMI difference occurred between the diabetic group (3) and its control group (*P* value: 1.48 × 10^−3^).

The results of age showed that the subjects of the (1) NAFLD-NASH group (*P* value: 4.6 × 10^−3^) and similarly of the (3) DM group (*P* value: 2.1 × 10^−3^) were significantly older in comparison to their control groups.

The results of blood pressure depicted in the different groups demonstrated an increase of systolic (SBP) and diastolic blood pressure (DBP) with the degree of NAFLD, displaying significant differences between (1) NAFLD-NASH and the relevant (1) control group (*P* value at SBP: 1.4 × 10^−2^ and *P* value at DBP: 4.0 × 10^−3^). Lipid status included total cholesterol (TC), low-density lipoprotein (LDL), high-density lipoprotein (HDL), and triglycerides (TG) (Figure 3) displayed significant differences only between (1) NAFLD-NASH group and the (1) control group (*P* value at TC: 2.4 × 10^−5^, *P* value at LDL: 4.0 × 10^−2^, and *P* value at HDL: 2.6 × 10^−6^). Merely, triglycerides showed significantly higher levels in the (1) NAFLD-NASH group (*P* value: 8.8 × 10^−12^) and in the (3) DM group (*P* value 1.17 × 10^−3^) as compared to respective control groups.

We analyzed traditional liver parameters aspartate transaminase (AST), alanine aminotransferase (ALT), AST/ALT ratio, and the Fasting Blood Glucose (FBG) (Figure 4).

Significant differences of ALT levels occurred in the (1) NAFLD-NASH group (*P* value: 2.3 × 10^−11^), the (2) NASH (*P* value: 3.37 × 10^−2^), and the (3) DM (*P* value: 9.3 × 10^−3^) as compared to control groups. AST levels displayed a significant difference between (1) NAFLD-NASH group and the (1) control group (*P* value: 1.8 × 10^−7^). FBG levels demonstrated significant differences in the (1) NAFLD-NASH group (*P* value: 2.6 × 10^−7^) and the (3) DM group (*P* value: 2.53 × 10^−3^) compared to their relevant control groups.

Finally, we considered serum uric acid (SUA) and serum ferritin as potential novel biomarkers for NAFLD diagnostics (Figure 5). The (3) DM group and respective (3) control were eliminated due to inconsistent data.

Both, SUA and serum ferritin were significantly different between the (1) NAFLD-NASH and the (1) control group (*P* value at SUA: 4.6 × 10^−5^ and *P* value at serum ferritin: 6.9 × 10^−3^).

### 4.2. Discussion

Despite of the huge amount of assessed data from 157 cohorts, our data analysis mostly revealed the already known and in clinical routine implemented results. Nonetheless, there are several remarkable results to be discussed.

First: our results demonstrated that no individual biomarkers are path-breaking, but a composition of biomarkers/biomarker grouping may be successful in clinical detection of NAFLD and its progression. Our analysis lies in (1) its scope of literature review, (2) the magnitude of cohorts included, (3) the number of parameters collected, and (4) its global approach [12–30, 41–83].

Second: overall, Europe [12–22] presented smaller cohorts than USA [23, 24] and Asia [25–30], due to larger population size and no geographical boundaries.

Gender and ethnicity inclusion failed because of no data.

Third: the selected parameters included standard NAFLD/NASH parameters such as liver enzymes and lipid profile. BMI and blood pressure were significantly lower in healthy control groups than in the disease group, which similarly confirmed the existing results. With respect to blood pressure, the outliers may present untreated hypertensive subjects. Furthermore, the results substantiate the theory of previous studies that the risk of NAFLD increases along with overweight and advanced age.

Differences of LDL between NAFLD subjects and their healthy control group as well as between diabetic subjects and their control group are lower than expected. In contrast, HDL levels of both the NAFLD healthy control group and the diabetic healthy control group are significantly higher as compared to their disease groups. This suggests a lower HDL level on the basis of disordered liver metabolism by NAFLD. Furthermore, the triglyceride level of NAFLD subjects was significantly higher than that of healthy controls. This may be the result of (a) reduced formation of VLDL and a disordered beta-oxidation, leading to lower LDL levels than expected, despite an increased triglyceride level, (b) triglyceride level of diabetic subjects is significantly higher than that of the NAFLD-NASH subjects (*P* value: 2.04 × 10^−2^) and suggests the effect of insulin resistance on triglyceride metabolism by increased adipolysis.

The FBG among diabetic subjects was higher than the FBG in the NAFLD group. In contrast, AST and ALT were increased by NAFLD, but diabetic subjects presented no pathological AST and ALT elevation as the healthy group. Our data analysis of ALT, AST, and FBG showed an increase attributable to the degree of NAFLD.

Fourth: parameters such as C-reactive protein (CRP), procollagen, or hyaluronic acid may not qualify as single biomarker because of their ubiquitary presence. Serum uric acid (SUA) differed significantly between the (1) healthy control and both liver disease groups. This finding supports the theory that patients with liver disease often receive diuretics or suffer from hepatic-renal syndrome, which both may lead to reduced renal SUA elimination and increased SUA levels [31].

An association between NAFLD and SUA is most plausible explained through the “two-hit” theory, which suggests that fat accumulation in hepatocytes presents the first hit and leads to an increased vulnerability of liver. Insulin resistance plays a crucial role in this vicious circle, which promotes lipolysis of the peripheral adipose tissue and increases the influx of free fatty acids into the liver. This insulin resistance leads to hyperinsulinemia, which increases the synthesis of uric acid and decreases its renal excretion. Although high levels of uric acid were a consequence of metabolic disorders, but it does not lead direct to NAFLD [32].

Other recent studies showed that serum uric acid is independently associated with NAFLD presence and development [33].

Furthermore, a possible gender effect can influence the development and progression of NAFLD and studies showed that the incidence of NAFLD increases after menopause [34].

Ferritin results showed a significant difference between the (1) healthy control and the (1) NAFLD-NASH group. This may be explained by inflammation leading to a serum ferritin increase due to macrophages' redistribution and by liver tissue damage due to macrophages redistribution [31]. The increased serum ferritin level may serve as independent predictor of liver damage in patients with NAFLD and is useable to identify patients at risk for NASH and fibrosis [35, 36]. Recent studies suggest the possibility of utilizing serum CK-18 and ferritin levels together to distinguish NASH from NAFLD [37].

Fetuin-A, as promising novel biomarker, appeared in the literature fragmentarily [38]. Recent studies showed that mRNA, protein expression, and the serum concentration of fetuin-A were increased in NAFLD patients. The gene expression of fetuin-A seems to be coregulated with key factors in the glucose and lipid metabolism. Furthermore, the oral antidiabetic metformin was able to decrease the fetuin-A level [39].

Gamma-glutamyl transpeptidase (GGT) was found rarely documented and could not be considered. Similarly adiponectin, resistin, various interleukins (ILs) were mentioned among others. Statistical analysis was impossible since no available data or too few studies were presented.

Though the traditional parameter results did not reveal surprising findings, they confirm the impact of lifestyle [40].

## 5. Conclusions

During our search on biomarkers we noticed, between the period of April to September 2011, a doubling of publications on NAFLD/NASH from 1,500 to over 3,000 articles in PubMed. Despite these tremendous research efforts, our findings did not display clinical innovation. The results also confirmed that at this point there is no one single biomarker detecting or differentiating NAFLD. Thus, our analysis suggests coordinated, standardized scientific research and the need for a collective look at biomarker groups and their link along with lifestyle, nutrition, exercise, genetics, and other factors. This applies accordingly also in clinical research. The topic of NAFLD/NASH is so complex and interdisciplinary that there is much space for further research in NAFLD development and its process of diagnosing, treatment, and prevention. In addition, our findings suggest the necessity of harmonized data acquisition and publishing as well as data visualization in meta-analyses for an effective NAFLD biomarker identification for future clinical practicability.

## Figures and Tables

**Table 1:** Definition of subtypes for data analysis.

(1) Control related to (1) NAFLD-NASH	(1) NAFLD-NASH contained healthy subjects (as defined and docusmented in the relevant studies), while the disease group consisted of NAFLD patients defined by the authors themselves.

(2) Control related to (2) NASH	(2) NASH comprised of NAFLD without NASH subjects, while NASH group consisted of NASH patients as defined by the authors themselves.

(3) Control related to (3) DM	(3) DM consisted of subjects without diabetes, while the disease group comprised of DM patients according to the publications.

(4) Control related to (4) MetSy	(4) MetSy—although initially considered for our statistical grouping—was omitted because of its inconsistent definition and inefficient available data.

**Table 2:** Overview of included studies with numbers of their cohorts and size, and statistical methodology [12–30, 41–83]. Reported results were displayed in mean or median. Cohorts below 60 subjects were included due to (1) overall many probands, (2) rare observed parameters, for example, CCT, AP, insulin, ferritin, and adiponectin, (3) small study because of small infrastructure of this country (e.g., European studies), and (4) rare documented diabetic probands of NAFLD studies.

Study	Total number of included cohorts of each study	Number of subjects of the smallest included cohort	Number of subjects of the largest included cohort	M: median A: mean MA: mean and median mixed	Remarks to studies with smaller cohorts than 60 subjects
Sun and Lü 2011 [72]	2	234	248	A	
Yasui et al., 2011 [71]	2	82	92	M	
Lee et al., 2010 [30]	3	1242	1276	A	
Xu et al., 2010 [29]	6	814	6077	MA	
Hwang et al., 2011 [28]	2	1613	3019	A	
Arase et al., 2011 [27]	1	5561	NA	A	
Thiruvagounder et al., 2010 [26]	4	61	76	A	
Xu et al., 2011 [25]	2	227	651	MA	
Tan et al., 2010 [70]	3	51	135	A	1
Caserta et al., 2010 [69]	2	74	498	M	
Ferreira et al., 2010 [68]	2	33	45	A	2
Park et al., 2011 [63]	2	145	311	A	
Sentinelli et al., 2011 [67]	2	239	346	M	
Kaelsch et al., 2011 [66]	2	56	71	A	1
de Luis et al., 2010 [21]	2	15	68	A	3
Alkhouri et al., 2010 [65]	3	11	36	MA	3
Barchetta et al., 2011 [64]	2	100	162	A	
Esteghamati et al., 2010 [62]	6	94	576	A	
Gupta et al., 2011 [61]	2	98	280	MA	
Kirovski et al., 2010 [60]	2	62	93	A	
Raszeja-Wyszomirska et al., 2010 [20]	2	14	48	A	3
Lee et al., 2010 [59]	2	24	25	A	2
Kilciler et al., 2010 [19]	2	54	60	MA	1, 3
Abdelmalek et al., 2010 [24]	2	84	224	A	
Qureshi et al., 2010 [58]	3	26	58	A	2
Adams et al., 2010 [57]	2	116	221	A	
Dongiovanni et al., 2010 [56]	2	202	346	A	
Harte et al., 2010 [18]	2	23	155	A	3
Younossi et al., 2011 [55]	2	39	40	A	2
Narciso-Schiavon et al., 2010 [53]	2	38	56	A	2
Oh et al., 2011 [48]	10	39	358	MA	1, 2
Söderberg et al., 2011 [17]	6	3	12	A	3
Tragher 2011	2	161	182	MA	
Aigner et al., 2010 [16]	2	27	124	A	3
García-Monzón et al., 2011 [15]	3	24	29	A	3
Neuschwander-Tetri et al., 2010 [23]	2	291	404	M	
Firneisz et al., 2010 [14]	3	23	82	A	3
Sumida et al., 2011 [51]	2	198	244	A	
Eguchi et al., 2011 [50]	3	74	375	A	
Ulitsky et al., 2010 [49]	2	52	201	A	1, 2
Williams et al., 2011 [48]	2	40	89	A	1, 2
Sokooian S 2010	3	45	102	A	1, 2
Hotta et al., 2010 [46]	4	64	578	A	
Tapan et al., 2010 [45]	2	31	65	MA	
Sokooian S 2010	2	102	188	A	2
Aller et al., 2010 [13]	2	15	51	A	3
Fierbinteanu-Braticevici et al., 2011 [12]	2	42	45	M	3
Rodriguez-Hernandez et al., 2010 [43]	4	29	229	A	4
Suzuki et al., 2010 [42]	2	22	62	A	2
Kalhan et al., 2011 [41]	3	11	25	A	2
Souza-Oliveira CPM 2010	2	45	86	A	2
Manousou et al., 2011 [22]	4	24	64	A	1, 3
Park et al., 2010 [82]	1	66	NA	A	
Sumida Y 2010	4	43	399	A	1, 2
Tanaka et al., 2011 [74]	1	55	NA	M	
Brunt et al., 2011 [80]	3	183	543	M	
Raszeja-Wyszomirska J 2010	1	104	NA	A	
Baba et al., 2011 [73]	1	165	NA	A	
Yilmaz Y 2010	2	56	58	A	1
Tsutsui et al., 2010 [77]	1	105	NA	A	
Verrijken et al., 2010 [76]	1	367	NA	A	
Akyildiz et al., 2010 [75]	2	91	104	A	
These 62 included articles contain 45 228 subjects of 157 cohorts				A = 75,8% M = 11,3% MA = 12,8%	

## Abbreviations

NAFLD: Nonalcoholic fatty liver disease
NASH: Nonalcoholic steatohepatitis
DM2: Diabetes type 2
IR: Insulin resistance
CVD: Coronary vascular disease
AFLD: Alcoholic fatty liver disease
ASH: Alcoholic fatty liver disease
MetSy: Metabolic syndrome
SD: Standard deviation
EDA: Exploratory data analysis
CI: Confidence interval
BMI: Body mass index
SBP: Systolic blood pressure
DBP: Diastolic blood pressure
TC: Total cholesterol
LDL: Low density lipoprotein
HDL: High density lipoprotein
ALT: Alanine transaminase
AST: Aspartate aminotransferase
FBG: Fasting blood glucose
MRI: Magnetic resonance imaging
HS: Hepatic steatosis
SUA: Serum uric acid
GGT: Gamma-glutamyl transpeptidase
ILs: Interleukins.
